# Autochthonous dengue in mainland France, 2022: geographical extension and incidence increase

**DOI:** 10.2807/1560-7917.ES.2022.27.44.2200818

**Published:** 2022-11-03

**Authors:** Amandine Cochet, Clémentine Calba, Frédéric Jourdain, Gilda Grard, Guillaume André Durand, Anne Guinard, Investigation team, Harold Noël, Marie-Claire Paty, Florian Franke, Investigation team, Michèle Auzet-Caillaud, Catherine Aventini, Alice Borel, Christiane Bruel, Anne Decoppet, Maurin Gossa, Mounira Krouk, Chloé Laboureyras, Alexia Mazza, Marie Mihoubi, Alexandra Muriel, Françoise Peloux-Petiot, Clément Piétin, Jérôme Raibaut, Laurent Saintillan, Camille Schmitt, Monique Travanut, Camille Vassal, Patricia Albert, Léa Bulmanski-Then, Cédric Cahuzac, Laura Catala, Aline Cot, Pierre-Marie Creton, Angélique Dubois, Jean Sébastien Dehecq, Isabelle Estève-Moussion, Fanny Gaillard, Christine Giraud, Olivier Glass, Vincent Lagarde, Aurélie Larrose, Catherine Lecerf, Radia Ould Larabi, Blandine Maes, Guylaine Peiffer, Christine Rico, Isabelle Rouvié-Laurie, Nicolas Roux, Giselle Santana, Nicolas Sauthier, Sarah Sellami, Stéphane Wagner, Betty Zumbo,, Estelle Balle, Lauranne Coiplet, Stéphanie Gaucher, Gabriel Leccia,, Delphine Morel,, Fabienne Jouanthoua,, Lorène Belkadi, Oriane Broustal, Cécile Durand, Damien Mouly, Gabriel Yubero, Adeline Riondel, Leila Bekheira, David Kelly, Isabelle Mertens-Rondelart, Miguel-Angel Sanchez-Ruiz, Yvan Souares, Nathan Yanwou,, Alexia Barbry, Thibaut Durand, Anne Ovize, Anaïs Soares,, Bénédicte Roquebert, Laura Verdurme,, Jean-Michel Mansuy, Hugues Aumaître,, Lionel Chanaud, Grégory L’Ambert, Yves-Marie Kervella,, Guillaume Lacour, Antoine Mignotte, Charles Tizon,, Anthony Biancarelli, Jean-Luc Maestracci, Barthélémy Pompa, Jean-Baptiste Santoni

**Affiliations:** 1 Santé publique France (French National Public Health Agency), Montpellier, France; 2 Santé publique France (French National Public Health Agency), Marseille, France; 3 French Armed Forces Biomedical Research Institute, National Reference Laboratory for Arboviruses, Marseille, France; 4 Unité des Virus Émergents (UVE: Aix-Marseille Univ-IRD 190-Inserm 1207), Marseille, France; 5 Santé publique France (French National Public Health Agency), Toulouse, France; 6 The members of the investigation team are listed under Collaborators; 7 Santé publique France (French National Public Health Agency), Saint-Maurice, France

**Keywords:** Dengue, mainland France, autochthonous, vector-borne disease

## Abstract

France faced an unusual situation of dengue transmission in 2022, with 65 autochthonous cases spread over nine transmission events by 21 October. This exceeded the number of cases observed during the entire period 2010 to 2021. Six of these events occurred in departments that had never experienced autochthonous dengue transmission. We provide an update of dengue surveillance data in mainland France in 2022. The multiplication of transmission events calls for continuous adaption of preparedness and response to arbovirus-related risks.

Because *Aedes albopictus* mosquito is expanding globally, increasing areas worldwide and in Europe have become at risk for *Aedes-*borne viruses. The French arbovirus surveillance system – deployed in mainland France since 2006 – has detected an increasing number of autochthonous transmissions of dengue virus (DENV), chikungunya virus (CHIKV) and Zika virus (ZIKV) since 2010 [1–5]. In 2022, the situation regarding DENV transmission in mainland France appears to be exceptional both in terms of number of transmission events and number of autochthonous cases (Figure 1).

**Figure 1:**
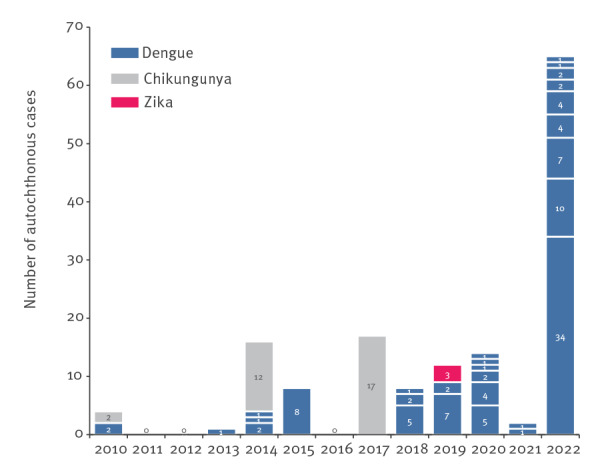
Distribution of arbovirus autochthonous events in mainland France, 2010–2022

Data for 2022 are not yet consolidated.

By 21 October 2022, the surveillance had identified 65 autochthonous cases of dengue fever, across nine transmission events. We describe here these events and their main characteristics.

## Arboviruses surveillance in France

Dengue is a notifiable disease year-round in France. The surveillance is enhanced from May to November, during the *Ae. albopictus* activity period [6], for timely implementation of vector control measures to prevent local virus transmission. Each year, regional health authorities launch the enhanced surveillance period with awareness campaigns targeting health professionals on diagnosis and reporting of dengue, but also chikungunya and Zika cases. In addition, Santé publique France Regional Offices review daily arbovirus diagnostic tests conducted in a nationwide network of laboratories to identify non-notified cases. Epidemiological investigations are carried out for each case, whether imported or autochthonous. An autochthonous case is defined as a case with no history of travel during the 2 weeks before symptom onset. Adapted vector control measures are then implemented in the different locations visited by the cases during their viraemic period (from 2 days before to 7 days after symptom onset).

The National Reference Centre for arboviruses (NRC) is in charge of laboratory confirmation of the first autochthonous cases in a local transmission event. According to the time between onset of symptoms and date of sampling (DPSO), the NRC performs RT-qPCR (DPSO < 7 days), ELISA using whole inactivated antigens (DPSO > 5 days), or both. When a RT-qPCR is positive, whole genome sequencing is attempted regardless of the quantification cycle (Cq) value, and virus isolation when Cq < 30. The NRC systematically tests for several flaviviruses (DENV, ZIKV and West Nile virus) and CHIKV. When an autochthonous case is identified, active case finding is immediately implemented in the area to determine the extent of local transmission: door-to-door survey in a 150 m to 250 m radius area, with fingertip blood sampling for suspected cases [7], health professional outreach and a press release for general population awareness. Any situation of autochthonous transmission is risk-assessed with regard to the safety of substances of human origin.

## Dengue transmission in mainland France in 2022

From 1 May to 21 October 2022, 217 imported cases of dengue were identified in mainland France. The majority of cases were imported from Cuba (n = 71), Ivory Coast (n = 16) and Mexico (n = 14). The number of imported cases in 2022 was similar to that observed in 2021 (164 cases), but considerably lower than in 2019 (n = 657) and 2020 (n = 834). In 2022, however, nine events of autochthonous dengue transmission were identified in mainland France, resulting in 65 total cases since the beginning of the surveillance period (Table).

**Table:** Main characteristics of dengue virus transmission events in mainland France, 2022 (n = 9)

Place of transmission			Number of autochthonous cases	Identification of the primary case	Serotype	Date of symptoms onset	
City	Department	Region				Earliest	Latest
Perpignan	Pyrénées-Orientales^a^	Occitanie	1	No	DENV-3	12 June	NA
Fayence	Var	Paca	7	No	DENV-1	20 June	22 July
Andrest/Rabastens-de-Bigorre	Hautes-Pyrénées^a^	Occitanie	4	Reunion Island, France	DENV-1	11 July	28 August
Saint-Jeannet/Gattières	Alpes-Maritimes	Paca	34	No	DENV-3	25 July	22 September
La Salvetat-Saint-Gilles	Haute-Garonne^a^	Occitanie	4	Democratic Republic of the Congo	DENV-3	14 August	20 August
Saint-Laurent-du-Var	Alpes-Maritimes	Paca	10	No	DENV-1	15 August	16 September
Montauban	Tarn-et-Garonne^a^	Occitanie	1	No	ND	30 August	NA
Toulouse	Haute-Garonne	Occitanie	2	No	DENV-3	15 September	21 September
Porto Vecchio region	Corse-du-Sud^a^	Corsica	2	No	DENV-3	20 September	20 September

DENV: dengue virus; NA: not applicable; ND: not determined; Paca: Provence-Alpes-Côte d’Azur.

^a^ First historic autochthonous circulation of *Aedes*-borne virus in the department.

The index case was notified by health professionals for six events. In addition, laboratory surveillance identified three transmission events. The size of the different autochthonous events ranged from 1 to 34 cases. The largest event occurred in the municipalities of Saint-Jeannet and Gattières, where respectively 23 and 11 cases were identified, belonging to a single transmission chain. All events occurred in suburban residential areas.

For seven of the nine events, the primary imported case was not identified. One local transmission chain was initiated by an imported case returning from Reunion Island (identified by laboratory-based surveillance), the other by a person arriving from the Democratic Republic of the Congo who did not seek medical attention during the symptomatic period (identified during the door-to-door survey). Neither of these two primary cases were notified by health professionals. The dengue serotype could be determined for eight events: five were serotype 3 (DENV-3), identified for the first time in mainland France, and three were serotype 1 (DENV-1). Whole genomes sequences were obtained for seven of the nine clusters. The DENV-3 sequence from the Corsica cluster could not be determined due to low viral load. The near complete sequences of the four remaining DENV-3 clusters were distinct from each other, suggesting distinct introductions in agreement with the available epidemiological data. Additional virological and phylogenetic analyses are still ongoing.

**Figure 2:**
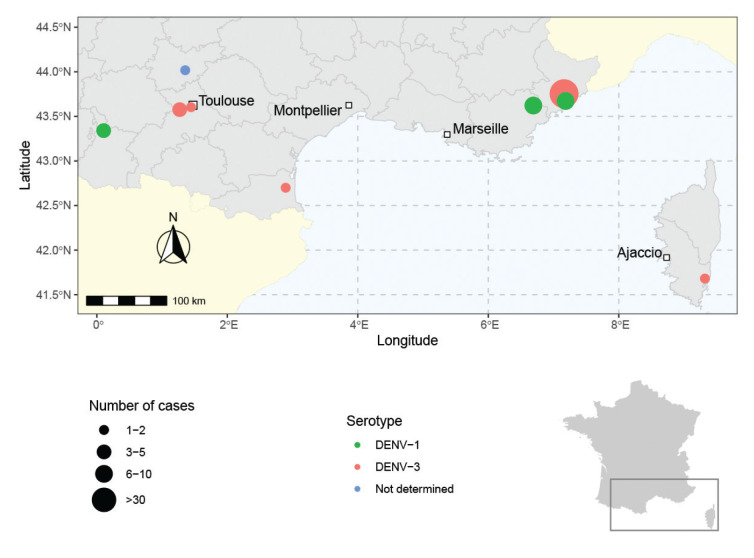
Dengue transmission events reported in mainland France, October 2022

Six of these events occurred in departments that had never previously experienced autochthonous transmission of an *Aedes*-borne virus, five in south-western France (Occitanie region) and one in Corsica (Figure 2). None of the cases presented with a severe form of dengue fever. The clinical manifestations of the 65 cases were mainly non-specific and included fever (98%), asthenia (78%), headache (75%), myalgia/arthralgia (62%), rash (43%) and retro-orbital pain (15%). Cases occurred throughout the surveillance period, from mid-June to the end of September.

Map created using GADM database of Global Administrative Areas, Version 4.1 available at www.gadm.org.

## Discussion

Given the history of colonisation by *Ae. albopictus*, events of autochthonous transmission of *Aedes*-borne viruses are expected in southern France [8] and several events have already been observed [2,4,5], as well as in other Mediterranean countries [9-11]. However, the evolving epidemiological situation of dengue in France in 2022 is exceptional, not only because of a large number of events, but also because the number of reported autochthonous cases (n = 65) exceeded that observed during the entire period 2010 to 2021 (n = 48) [2,12]. Among the nine events reported this year, one is the largest ever documented in Europe, with 34 cases of dengue identified on 21 October 2022 (Figure 2). Moreover, six events took place in departments where such transmission had never been described (south-western France, Corsica).

We assume that the increase in cases is not a surveillance artefact because surveillance was maintained during the COVID-19 outbreak. The drivers of arbovirus transmission are undoubtedly multiple but are mainly influenced by the interactions between vector populations, virus strains and the global environment [13]. Environmental conditions thus have a major impact on the efficiency of the vector system as well as on vector density and host–vector contacts [14,15]. Spring and summer have been especially warm in 2022 [16], which promoted vector activity and transmission efficacy of dengue virus [17]. Adaptation between viral and vector genotypes is also a major factor in transmission, and the occurrence of numerous episodes involving DENV-3 raises questions. Further research is needed to better characterise determinants (climatic, socio-economic, environmental) for local transmission events and their extension. Such analysis needs to take into account all autochthonous transmission events observed in France since 2010.

## Conclusion

France is the only European country to have declared autochthonous dengue cases this year [18]. The French surveillance system appears to be sufficiently sensitive to detect autochthonous transmission of arboviruses and efficient to limit their spread. However, the multiplication and the geographical extent of transmission events are challenging its sustainability. Ensured sustainability of this surveillance requires promotion of main stakeholder involvement through (i) consolidation of the network of reporting laboratories, (ii) raising awareness among patients to seek medical consultation for influenza-like illness without respiratory symptoms, especially in the context of negative COVID-19 results and (iii) orientation of health professionals to the diagnosis and reporting of arboviral diseases.

## The members of the Investigation team were;

Michèle Auzet-Caillaud, Catherine Aventini, Alice Borel, Christiane Bruel, Anne Decoppet, Maurin Gossa, Mounira Krouk, Chloé Laboureyras, Alexia Mazza, Marie Mihoubi, Alexandra Muriel, Françoise Peloux-Petiot, Clément Piétin, Jérôme Raibaut, Laurent Saintillan, Camille Schmitt, Monique Travanut, Camille Vassal (Agence régionale de santé Provence-Alpes-Côte d’Azur); Patricia Albert, Léa Bulmanski-Then, Cédric Cahuzac, Laura Catala, Aline Cot, Pierre-Marie Creton, Angélique Dubois, Jean Sébastien Dehecq, Isabelle Estève-Moussion, Fanny Gaillard, Christine Giraud, Olivier Glass, Vincent Lagarde, Aurélie Larrose, Catherine Lecerf, Radia Ould Larabi, Blandine Maes, Guylaine Peiffer, Christine Rico, Isabelle Rouvié-Laurie, Nicolas Roux, Giselle Santana, Nicolas Sauthier, Sarah Sellami, Stéphane Wagner, Betty Zumbo (Agence régionale de santé Occitanie) ; Estelle Balle, Lauranne Coiplet, Stéphanie Gaucher, Gabriel Leccia (Agence régionale de santé Corse) ; Delphine Morel (Agence régionale de santé Grand-Est) ; Fabienne Jouanthoua (Agence régionale de santé Nouvelle-Aquitaine) ; Lorène Belkadi, Oriane Broustal, Cécile Durand, Damien Mouly, Gabriel Yubero, Adeline Riondel, Leila Bekheira, David Kelly, Isabelle Mertens-Rondelart, Miguel-Angel Sanchez-Ruiz, Yvan Souares, Nathan Yanwou (Santé publique France); Alexia Barbry, Thibaut Durand, Anne Ovize, Anaïs Soares (Biomnis) ; Bénédicte Roquebert, Laura Verdurme (Cerba); Jean-Michel Mansuy (CHU Toulouse), Hugues Aumaître (CH Perpignan); Lionel Chanaud, Grégory L’Ambert, Yves-Marie Kervella (EID-Méditerranée) ; Guillaume Lacour, Antoine Mignotte, Charles Tizon (Altopictus); Anthony Biancarelli, Jean-Luc Maestracci, Barthélémy Pompa, Jean-Baptiste Santoni (Collectivité de Corse).
